# Prognostic factors in surgically treated tongue squamous cell carcinoma in stage T1‐2N0‐1M0: A retrospective analysis

**DOI:** 10.1002/cam4.7016

**Published:** 2024-02-24

**Authors:** Wenxi Wang, Yuxiang Wang, Wenhui Zeng, Xubin Xie, Chen Li, Qin Zhou, Liangfang Shen

**Affiliations:** ^1^ Department of Oncology, Xiangya Hospital Central South University Changsha Hunan Province China; ^2^ Department of Radiation Oncology Fourth Hospital of Hebei Medical University Shijiazhuang Hebei Province China

**Keywords:** lymph node, prognosis, recurrence, surgery, survival, tongue squamous cell carcinoma

## Abstract

**Purpose:**

The study aimed to retrospectively identify the prognostic factors of surgically treated primary tongue squamous cell carcinoma (TSCC) cases and assess the benefits of surgical neck lymph node dissection (LND) in early‐stage cancer.

**Methods:**

Patients with primary TSCC with pT1‐2N0‐1M0 stage without distant metastasis who were treated with surgery during 2014–2016 at Xiangya Hospital, Central South University were included. Univariate and multivariate Cox models were constructed to explore prognostic factors of overall survival (OS), disease‐free survival (DFS), and local recurrence‐free survival (LRFS). Sub‐group analysis was used to assess the effect of adjuvant therapy and the prognostic value of LND for the early‐stage patients.

**Results:**

In total, 440 patients met the inclusion criteria. During the follow‐up period, the 5‐year OS, DFS, were 84.4% and 70.0%, respectively. Univariate analysis showed that TNM stage, lymphovascular invasion (LVI), and/or perineural invasion (PNI), pathological differentiation, etc. were significant predictors of OS and DFS. Multivariate analysis showed that TNM stage and the degree of pathological differentiation were independent prognostic factors for all outcomes. Besides, the number of cervical LND could independently predict both DFS and LRFS while LVI/PNI were associated with DFS. And high‐quality neck LND (≥30) significantly improved DFS and LRFS for patients of pT1cN0M0 or stage I as compared to those without LND.

**Conclusions:**

TNM stage and pathological differentiation were crucial prognostic factors for postoperative patients with TSCC. Notably, high‐quality cervical LND was beneficial for the improvement of DFS and LRFS for patients of pT1cN0M0 or stage I.

## 1 INTRODUCTION

Data from GLOBALCAN indicates that an estimated 377,713 people had oral or lip cancer during 2020, among which around 46% (177,757) patients died.^1^ Although oral cancer is not among the highest cancer‐associated mortality, facial disfigurement, loss of speech, and eating function caused by the disease and its treatment can cause profound morbidity and negative effects on patient quality of life. Established risk factors of oral cancer include tobacco smoking^2^ and alcoholism.^3^ Betel nut chewing has been identified as a major risk factor for oral cancer incidence in areas with high betel nut consumption, such as the Indian subcontinent and Taiwan.^4^ There is also a high incidence of oral cancer in Hunan province, China, where betel nut is commonly used.

Oral tongue squamous cell carcinoma (TSCC), which arises at the anterior two thirds of the tongue, is the most common malignancy diagnosed within the oral cavity.^5^ Treatment of TSCC includes comprehensive management including surgery with or without radiotherapy and chemotherapy. Treatment approaches are considered based on multiple outcome‐related factors, such as patient age, tumor stage, lymphovascular invasion (LVI), perineural invasion (PNI), positive surgical margins, and lymph node extracapsular extension.^6^, ^7^, ^8^, ^9^, ^10^ According to the 2022 National Comprehensive Cancer Network (NCCN) guidance, surgery remains the major treatment option, including for some patients with III–IV stage, reflecting its governing position in oral cancer therapy.^11^

Lymph node metastasis is the most well‐recognized prognostic factor in negative oral cancer prognosis.^12^ Others^13^ have demonstrated that pN stage plays a predominant role in outcomes of patients with TSCC treated with primary surgery and appropriate adjuvant therapy. However, the issue of lymph node dissection (LND) in oral cancer patients in early stage has been the subject of much discussion.^14^ Tumor grade, depth of invasion, and subsite are crucial factors for assessing the benefit of elective neck dissection in such early‐stage oral cancer cases.^15^ The choice of whether and how to perform lymphatic dissection, especially for patients with oral cancer T1N0M0 merits further investigation.

In the present study, we analyzed data from 440 primary TSCC patients with pT1‐2N0‐1M0 stage treated at Xiangya Hospital during 2014–2016 to identify the predictors of patient outcomes and further explore the effects of LND in early‐stage cancer.

## 2 METHODS

### 2.1 Patient selection

Consecutive patients with TSCC who were categorized as code C02 according to the International Classification of Disease for Oncology, 10th revision (ICD‐10) were selected from the medical record system of Xiangya Hospital, Central South University, Hunan, China. The inclusion criteria were as follows: newly diagnosed TSCC patients with stage pT1‐2N0‐1M0 who underwent surgical resection from January 1, 2014 to December 31, 2016. Exclusion criteria were as follows: patients who were diagnosed with tongue base cancer; histological diagnosis not consistent with squamous cell carcinoma; patients who did not undergo surgery in the study center hospital or had received treatment prior to reporting to the study center; patients who had secondary cancer or patients who had distant metastasis; patients who had postoperative chemotherapy alone; patients who did not have complete clinical records in the hospital medical record system. A total of 440 consecutive patients who fulfilled the abovementioned criteria were included retrospectively in the study. The study protocol was reviewed and approved by the Ethical Review Committee of the Xiangya Hospital Central South University, Hunan, China (202212296).

### 2.2 Data collection

Patient characteristics including age, gender, admission time, surgical information, pathological diagnosis, and application of adjuvant therapy if any and its type, were recorded. TSCC TNM staging was categorized based on 7th American Joint Committee on Cancer (AJCC) classification system.

### 2.3 Follow‐up protocol

Telephonic follow‐up was primarily used to acquire information from patients and their caregivers, including their current condition, further treatment received, concomitant disease conditions, relapse, salvage treatment, and death. For those patients who could not be contacted, information was collected from hospital outpatient records and imaging records to note any disease recurrence. The date of surgery at Xiangya Hospital was considered the baseline event for the study, and the endpoints varied for different outcomes. For overall survival (OS), death, and the last follow‐up were considered as endpoint. For disease‐free survival (DFS), the endpoints were death, recurrence, or the last follow‐up. For local recurrence‐free survival (LRFS, including local and regional lymph node recurrence) and distant metastasis‐free survival (DMFS), the endpoints were last follow‐up/death or local‐regional recurrence or distant recurrence, respectively. The follow‐up duration was counted in months from the date of primary surgery to August 2021 or the date of last known event.

### 2.4 Statistical analysis

Data were analyzed using SPSS (v22.0, IBM Corporation, USA) and R (version 4.3.0). Univariate and multivariate Cox analyses were applied to evaluate the potential prognostic factors related to survival outcomes including OS, DFS and LRFS. Kaplan–Meier survival analysis and log‐rank test were used to compare various survival curves. Then, we divided patients into groups according to T or N stage and sub‐group analysis was performed to compare survival outcomes of patients with or without adjuvant therapy, or with various numbers of LND. Count data were analyzed by chi‐square test. The results are presented as hazard ratios (HR) with 95% confidence interval (95% CI), and *p* < 0.05 was recognized as statistically significant.

## 3 RESULTS

### 3.1 Clinical characteristics

A total of 440 postoperative patients with TSCC were included in this study. The demographic characteristics of the enrolled cases are shown in Table 1. The median age was 50 (range 22–83) years. 393 (89.3%) patients were male, and 52 (11.8%) cases had weight loss before admission. More than 85% of patients had habits of smoking or drinking, or chewing betel nut.

**TABLE 1:** Clinical characteristics of the study sample (pT1‐2N0‐1M0, *n* = 440).

Items	Groups	Cases		Items	Groups	Cases	
		*n*	%			*n*	%
Gender	Male	393	89.3	LND	Yes	393	89.3
	Female	47	10.7		No	47	10.7
Age	50 (22–83)	–	–	Number of LND	15 (1–173)	–	–
Site of lesion	Left	227	51.6		No	47	10.7
	Right	213	48.4		<15	172	39.1
Tobacco	No	115	26.1		15–29	136	30.9
	Yes	309	70.2		≥30	85	19.3
	Quit >1 year	16	3.6	Differentiation	Well	295	67.0
Alcohol	No	174	39.5		Middle	129	29.3
	Yes	104	23.6		Poor	16	3.6
	Occasional	154	35.0	T stage	T1	267	60.7
	Quit >1 year	8	1.8		T2	173	39.3
Betel nut	No	143	32.5	N stage	N0	341	77.5
	Yes	217	49.3		N1	99	22.5
	Occasional	49	11.1	TNM stage	I	227	51.6
	Quit >1 year	31	7.0		II	114	25.9
Lost weight	No	365	83.0		III	99	22.5
	Yes	52	11.8	Adjuvant therapy	No	398	90.5
	No record	23	5.2		RT	17	3.9
LVI/PNI	No	388	88.2%		CRT	25	5.7
	Yes	10/42	2.3%/9.5%		–	–	–

Abbreviations: CRT, chemoradiotherapy; LND, lymph node dissection; LVI, lymphvascular invasion; PN1, perineural invasion; RT, radiotherapy.

Among the 393 (89.3%) patients who underwent neck dissection, 172 (39.1%), 136 (30.9%), and 85 (19.3%) patients had dissected lymph node numbers of <15, 15–29, or ≥30, respectively. There were 16 (3.6%), 129 (29.3%), and 295 (67%) patients who were pathologically diagnosed as poorly, moderate, or well‐differentiated, respectively. 49 (11.1%) patients had LVI and/or PNI. Furthermore, 267 (60.7%), 173 (39.3%) patients were identified as T1 and T2 stages after surgery. The proportions of N0, N1 were 341 (77.5%), 99 (22.5%), respectively. And 227 (51.6%) patients were pathological stage I, 114 (25.9%) were stage II, 99 (22.5%) were stage III. In addition to surgery, 42 (9.6%) patients underwent postoperative adjuvant therapy, including radiotherapy monotherapy (17 [3.9%]) and combined chemoradiotherapy (CRT) (25 [5.7%]).

The median follow‐up time was 61 months (range 0.5–91 months). Among all the patients, 379 cases survived and 61 cases died, of which 53 patients died from primary tumor, 4 patients died from secondary tumors, 3 patients died of other reasons like cardiovascular diseases, and 1 died of an undefined reason. In addition, a total of 119 cases had TSCC‐related progression after surgery, including 107 cases with loco‐regional recurrence only, 8 cases with distant metastasis (DM) only, 4 cases with both local‐regional failure and DM. One patient with lost follow‐up were defined as survival of 0.5 months after surgery.

### 3.2 Prognostic factors in postoperative TSCC patients

#### 3.2.1 OS and DFS

The 1‐, 3‐, and 5‐year OS for the study group was 94.8%, 86.1%, and 84.4%, respectively (Figure S1). The DFS at 1, 3, and 5 years was 82.0%, 73.8%, and 70.0%, respectively (Figure S1). The results of univariate and multivariate Cox analyses are presented in Table S1 and Table 2 respectively, showing the prognostic factors of OS and DFS. According to univariate Cox analysis, more advanced T (2.186 [1.321–3.619], *p* = 0.002), N (2.717 [1.631–4.527], *p* < 0.001) and TNM stage (*p* < 0.001), poor pathological differentiation (4.374 [1.836–10.419], *p* = 0.001), existence of LVI/PNI (2.386 [1.268–4.491], *p* = 0.007) were associated with worse OS (Table S1). Furthermore, T (1.507 [1.070–2.122], *p* = 0.019), *N* (1.613 [1.110–2.346], *p* = 0.012), TNM stage (*p* = 0.008), the number of LND (*p* = 0.087), degree of pathological differentiation (*p* < 0.001), LVI/PNI (2.101 [1.337–3.301], *p* = 0.001) were also shown to be associated with DFS. (Table S1). However, the history of tobacco, alcohol, or betel nut consumption were not significantly related to OS and DFS in this study (data not shown).

**TABLE 2:** Multivariate Cox regression analysis of predictors of OS and DFS for postoperative pT1‐2N0‐1M0 TSCC patients.

Items	Factors	Groups	HR (95% CI)	*p*
OS	Differentiation	Well	1	0.023
		Middle	1.329 (0.762–2.318)	0.316
		Poor	4.092 (1.707–9.810)	0.002
	TNM	I	1	<0.001
		II	3.055 (1.582–5.901)	0.001
		III	4.042 (2.146–7.611)	<0.001
DFS	LND	≥30	1	0.001
		No	0.473 (0.270–0.830)	0.009
		<15	0.551 (0.312–0.972)	0.04
		15–29	0.232 (0.113–0.477)	<0.001
	Differentiation	Well	1	0.001
		Middle	1.767 (1.212–2.577)	0.003
		Poor	3.462 (1.672–7.167)	0.001
	TNM	I	1	0.002
		II	1.729 (1.123–2.663)	0.013
		III	2.170 (1.384–3.401)	0.001
	LVI/PNI	No	1	–
		Yes	1.581 (0.981–2.548)	0.060

Abbreviations: DFS, disease‐free survival; OS, overall survival; TSCC, tongue squamous cell carcinoma.

Multivariate Cox analysis incorporated and analyzed four factors for OS (age, TNM stage, degree of pathological differentiation, LVI, and/or PNI) and DFS (the number of LND, TNM stage, degree of pathological differentiation, LVI, and/or PNI) whose *p* < 0.1 at univariate Cox analysis. The results revealed that poor pathological differentiation (4.092 [1.707–9.810], *p* = 0.002) and late TNM stage (*p* < 0.001) were independent risk factors for OS. In addition, the number of LND (*p* = 0.001), TNM stage (*p* = 0.002), and degree of pathological differentiation (*p =* 0.001) could independently predict DFS (Table 2), while the existence of LVI/PNI was a non‐significant to negatively predictor of DFS (1.581 [0.981–2.548], *p* = 0.06). These data indicated that TNM stage and degree of pathologic differentiation were main factors predicting OS and DFS.

#### 3.2.2 LRFS

LRFS recognized local recurrence at the primary tumor and regional recurrence of lymph node as the endpoint. The 1‐, 3‐, and 5‐year LRFS and distant metastasis free survival (DMFS) were 83.8% and 98.3%, 77.4% and 96.9%, 74.5% and 96.9%, respectively (Figure S2). Most frequently, recurrence occurred within the first year after surgery. Univariate Cox analysis showed that the number of LND (*p* = 0.077), pathological differentiation (*p* < 0.001), and LVI and/or PNI (1.897 [1.143–3.151], *p* = 0.013) were predictive factors for LRFS (Table S2). The multivariate Cox analysis included three factors for LRFS (TNM stage, degree of pathological differentiation, LVI, and/or PNI). We found that the TNM stage (*p* < 0.05), the number of LND (*p* = 0.002) and pathological differentiation (*p* = 0.001) were all independent risk factors for LRFS (Table 3).

**TABLE 3:** Multivariate analysis of predictors of LRFS for pT1‐2N0‐1M0 TSCC patients.

Factors	Groups	HR (95% CI)	*p*
LND	≥30	1	0.002
	No	0.489 (0.274–0.872)	0.015
	<15	0.528 (0.291–0.957)	0.035
	15–29	0.236 (0.109–0.508)	<0.001
Differentiation	Well	1	0.001
	Mmiddle	1.618 (1.072–2.444)	0.022
	Poor	4.431 (2.168–9.053)	<0.001
TNM	I	1	0.020
	II	1.642 (1.039–2.595)	0.034
	III	1.887 (1.150–3.097)	0.012

Abbreviations: LND, lymph node dissection; TSCC, tongue squamous cell carcinoma.

Since only 12 patients had distant metastasis, we did not include this factor in the univariate and multivariate analysis for DMFS.

### 3.3 Sub‐group analysis of adjuvant therapy

**FIGURE 1:**
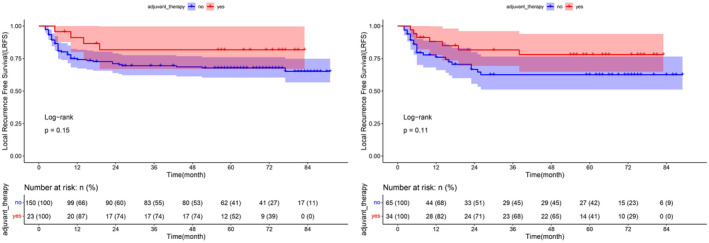
LRFS in patients with pT2 and pN1 TSCC after surgery with or without adjuvant therapy. LRFS, local recurrence‐free survival; TSCC, tongue squamous cell carcinoma.

The NCCN guidelines recommend adjuvant radiotherapy or chemoradiotherapy for patients with advanced clinical features like LVI/PNI, locally advanced stage, etc. In this study, there were 51.6% patients with stage I, 25.9% with stage II and 22.5% with stage III disease. Under univariate Cox analysis, postoperative adjuvant therapy was not significantly associated with OS, DFS, or LRFS (all *p* > 0.05, Tables S1 and S2). However, as disease stage is an important correlate of treatment choice and outcome, sub‐group analysis was performed to explore the effect of adjuvant therapy. We first selected patients with pT2 and pN1, then by comparing patients with or without postoperative adjuvant therapy, we found that RT/CRT tended to increase LRFS for patients of pT2 or pN1 (Table S3, Figure 1). Whereas in the remaining analysis, patients with or without postoperative adjuvant therapy showed no significant differences in OS, DFS, or LRFS, (*p* > 0.05, Table S3).

### 3.4 Effect of cervical LND for pT1cN0‐1M0 or stage I TSCC patients

**FIGURE 2:**
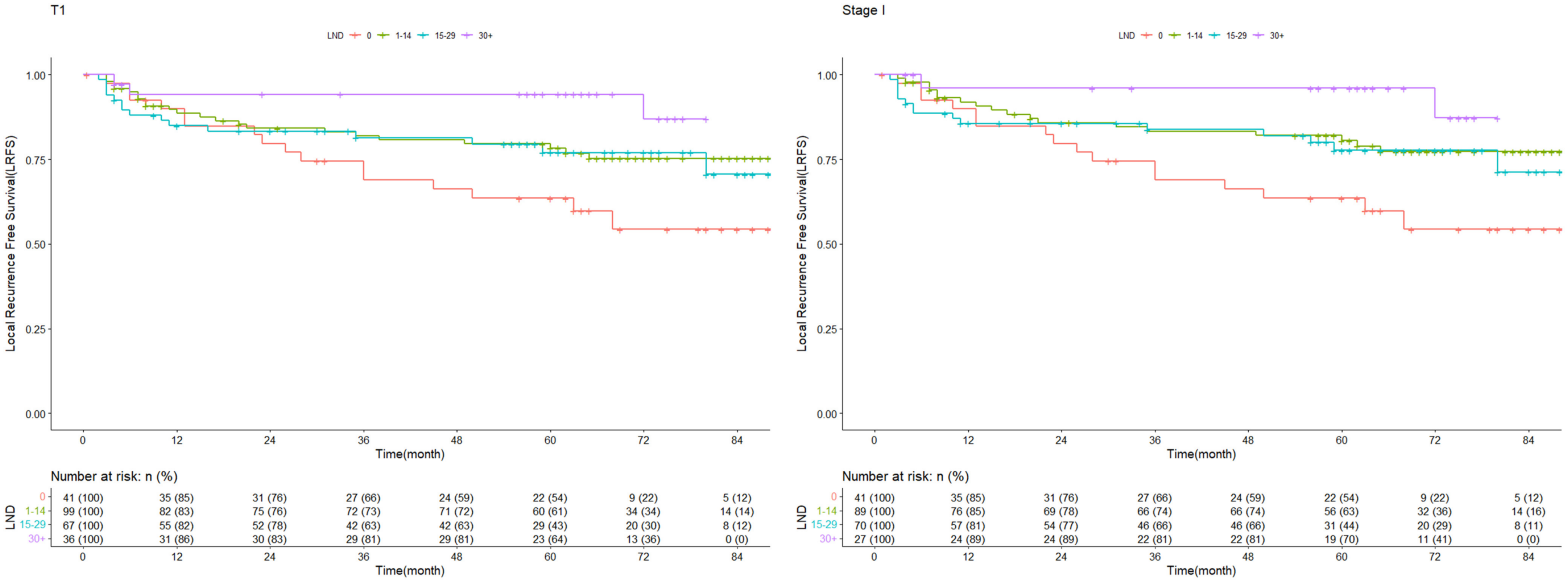
LRFS in patients with pT1cN0 and stage I TSCC after surgery with or without LND. LRFS, local recurrence‐free survival; TSCC, tongue squamous cell carcinoma.

In this study, 41 out of 267 (15.4%) patients in pT1 stage and 6 out of 173 (3.5%) patients in pT2 stage did not undergo neck LND. To explore the function and effects of LND, we first collected patients of pT1cN0 or stage I. Then we categorized patient into four groups according to the number of LND: 0 (without LND), <15, 15–29, and ≥30. For pT1cN0 patients, we found that LRFS was significantly different between patients with or without LND (Table 4, Figure 2, *p* < 0.05). Furthermore, the DFS and LRFS for those who had ≥30 LND was significantly higher than that of those without LND and tended to be higher than those <15 or 15–29 cervical LND (*p* = 0.007, 0.106, 0.067, respectively for DFS and *p* = 0.003, 0.088 and 0.086 respectively for LRFS, Table 4). However, there was no significant improvement for OS in pT1cN0 patients with or without LND (Table S4).

**TABLE 4:** DFS and LRFS in T1cN0M0 or stage I postoperative TSCC patients with or without LND.

	LND	Cases	DFS			*p*	LRFS			*p*
			1‐year	3‐year	5‐year		1‐year	3‐year	5‐year	
T1cN0M0	No	41	89.9	69.0	63.5	0.072	89.9	69.0	63.5	0.028
	<15	99	85.6	79.1	75.7		88.6	81.9	78.3	
	15–29	67	84.9	77.9	71.9		84.9	81.4	77.0	
	≥30	36	94.2	74.2	90.6		94.2	94.2	94.2	
Stage I	No	41	89.9	69.0	63.5	0.023	89.9	69.0	63.5	0.022
	<15	89	89.7	81.3	78.6		91.9	83.3	80.6	
	15–29	70	84.1	79.2	73.4		85.6	83.8	77.7	
	≥30	27	96.0	96.0	96.0		96.0	96.0	96.0	

Abbreviations: DFS, disease‐free survival; LND, lymph node dissection; LRFS, local recurrence‐free survival; TSCC, tongue squamous cell carcinoma.

For patients with stage I TSCC, the LRFS and DFS were significantly different between patients with various numbers of LND (Table 4, Figure 2, *p* < 0.05). The LRFS was lower in those without cervical LND than those with <15, and ≥30 LND (*p* = 0.026, 0.005, respectively). Furthermore, the 5‐year DFS was worse for patients without cervical LND compared to those with ≥30 of LND (*p* = 0.005). However, similar results were not observed when comparing OS (Table S4).

By analyzing the pattern of recurrence in patients of pT1cN0 or stage I. we also found that the patients in the group without LND had more frequent local and lymph node recurrence (*p* = 0.009, *p* = 0.042). When LND was ≥30, those patients not only had the lowest ratio of regional lymph node recurrence but also the least local recurrence. (Table S5, *p* < 0.05).

## 4 DISCUSSION

This was a single‐center retrospective study that included a large number of patients with TSCC collected over the course of a relatively short period of time (2014–2016). Our data were largely consistent with regional characteristics regarding the prevalence of environmental risk factors noted in Hunan Province, China.^16^, ^17^, ^18^ More than 85% of the patients in our study had a history of betel nut consumption, smoking, or drinking. The 5‐year OS, DFS, and LFRS of the whole group in T1‐2N0‐1 were 84.4%,70.0%, and 74.5%, respectively. Most of the recurrence events happened in the first 2 years (76.6%) which indicates that regular follow‐up is critical. Earlier retrospective studies of postoperative TSCC have reported slightly lower survival rates. Ganly et al.^19^ analyzed 216 patients with postoperative tongue cancer in the early stage over 20 years and found the 5‐year OS and RFS were 79% and 70%, respectively. Similarly, Fridman et al^20^ analyzed 1257 patients of oral cavity squamous cell carcinoma in stage T1‐2N0 (T1: 36.7%, T2: 63.3%) after surgery and reported the 5‐year OS, DFS, disease‐specific survival (DSS), and LRFS were 75%, 73%, 83%, and 79%, respectively. Besides, Daniell et al.^21^ also reported a similar distribution (T1: 53%, T2: 35%) with a 69% 5‐year OS and a 75% 5‐year freedom from loco‐regional failure of 258 patients with postoperative TSCC from 2007 to 2016. In the present study, 89.3% of the patients received LND, with 50.2% having >15 LND. The rate of postoperative pathological positive margins or LVI/PNI were relatively low (2.3% [10/440] cases and 9.5% [42/440], respectively). Overall, the results of this study can be considered representative of the outcomes of radical resection in TSCC populations with a high prevalence of betel nut chewing and thus provide valuable clinical reference.

The TNM stage is one of the main prognostic factors of TSCC patients after surgery.^22^, ^23^, ^24^ In accordance, the present study found that pTNM stage was an independent prognostic factor of OS, DFS, and LRFS. Besides, pT and pN stage also have been reported as independent risk factors of OS and disease‐specific survival for patients with oral cancer,^13^, ^25^ which is consistent with our finding that stage pT and pN were associated with OS and DFS. Furthermore, pN stage has also been found to be predictive of OS in TSCC after surgery^19^, ^26^ and emerged as a key predictor of survival outcomes. In the present study, patients with pN0 and pN1 stage had 5‐year OS of 88.4% and 69.9%, with the 5‐year DFS of 73.5% and 57.4%, respectively (data not shown). These results revealed that advanced stage at time of surgery predicted worse outcomes and highlighted the need for early diagnosis and timely treatment.

A high‐quality cervical LND involve at least 18 lymph nodes for the patients of oral cancer.^27^ In the whole group, the median number of LND was 15. We divided patients into four groups according to numbers of LND (0 [without LND], <15, 15–29, and ≥30) and performed multivariate analysis. The results showed that LND was found to be an independent predictor for DFS and LRFS. We also noted that the DFS and LRFS of patients with ≥30 LND was significantly higher than those without LND. However, the number of LND did not affect OS. These findings suggest that for TSCC patients of stage T1‐2N0‐1, high quality of LND may help to avert early disease recurrence. Elective dissection of cervical lymph nodes during surgical management of early‐stage oral cancer has been a subject of debate. The ASCO clinical practice guidelines suggest that LND can be performed for oral cancer patients with T1N0M0 along with close ultrasound surveillance.^27^ Others have proposed that observation rather than dissection or adjuvant therapy should be recommended for patients with T1‐2N0 oral cancer without neural or vascular invasion.^28^ A meta‐analysis showed that for patients with T1‐2N0 OTSCC, LND can significantly prevent local recurrence and thus increase disease specific survival but not OS.^29^ Another meta‐analysis including 1250 patients (7 randomized controlled trials) in early‐stage OSCC with clinical N0 patents found that in comparison to observation, elective neck dissection could significantly improve OS and DSS, and significantly reduce lymph node recurrence.^30^ Furthermore, the number of LND is also important for better outcome. Cheng et al.^31^ noted that the survival outcome was significantly higher in the group with ≥37 retrieved lymph nodes for patients of T1‐2N0M0 oral cancer. In the present study, we found that the DFS and LRFS were significantly higher in patients with stage pT1cN0M0 or stage I when LND ≥30, whereas those without LND had the lowest DFS and LRFS, and higher local and regional recurrence rates. To further explore the correlated of recurrence, we confirmed the findings with chi‐square test which showed that when LND ≥30, both local and lymph node recurrence was lower as compared to those without LND. However, OS was not improved by LND, which may be due to the fact that disease recurrence did not cause death for patients of stage pT1cN0 and stage I, and the 5‐year OS in these patients remained as high as over 90%. Above all, these findings indicated that not only for patients with TSCC in stage T1‐2N0‐1 but also for those with stage pT1cN0 or stage I, high quality of LND (≥30) was beneficial for DFS and LRFS and thus should be considered at the first surgery.

The NCCN guidelines describe LVI/PNI as adverse pathologic and prognostic features.^32^ The presence of LVI/PNI is associated with a worse prognosis in TSCC,^9^, ^13^, ^25^, ^33^, ^34^, ^35^ especially for those who had multifocal intra‐ and peritumoral PNI.^36^ LVI was also reported be associated with cervical lymph node metastasis and loco‐regional recurrence.^37^ Patients with pN0 stage oral cancer^38^ or TSCC^39^ with LVI/PNI show worse loco‐regional control and OS, suggesting the need for further adjuvant therapy. In this study, the incidence of LVI and PNI was 2.3% and 9.5%, respectively. The univariate analysis results showed that LVI/PNI predicted worse OS, DFS and LRFS. LVI/PNI also tended to decrease DFS in the multivariate Cox analysis (*p* = 0.060). The insignificant result may be due to the substantial difference in sample size between the two groups but the trend suggested that LVI/PNI could be a predictor of recurrence outcome, and merits further investigation.

The benefits of postoperative adjuvant therapy in TSCC are a subject of research. The NCCN guidelines recommend that for patients with T1‐2N0 oral cancer without risk factors, follow‐up after surgery is feasible.^5^ Several studies have demonstrated insignificant benefits of adjuvant postoperative radiotherapy in early‐stage TSCC.^20^, ^40^ While other studies found that OS of patients with oral cancer (T1‐2N1) benefited from postoperative adjuvant therapy when the number of LND was <18^41^ or when lymph node ratio (LNR) was >5.5% in OTSCC patients.^42^ Fridman et al^20^ also reported that adjuvant therapy significantly improved survival when there were positive/close margin for patients with T1–2N0M0 oral cavity squamous cell carcinoma. In this study, no survival improvement was notable for the pT1‐2N0‐1 TSCC patients with adjuvant therapy as a group, which may be related to the rare incidence of positive margins, high ratio of LND, less frequent LVI/PNI and the distribution of TNM stage categories among patients who underwent adjuvant therapy. Hence, we performed sub‐group analysis and observed a trend for pT2 and pN1 patients which confirmed that adjuvant therapy may improve LRFS, reflecting the potential function of postoperative therapy for patients with more advanced stage disease. Similarly, another study also reported that postoperative radiotherapy may benefit patients with oral cancer in stage T1‐2N1.^43^ Therefore, adjuvant therapy may be an important factor for improving survival outcome, especially when lymph node metastasis exists.

In this study, pathological differentiation emerged as the other independent factor that predicted OS, DFS, and LRFS in patients of T1‐2N0‐1M0 TSCC. Poorer differentiation was related with worse survival and more frequent recurrence. Several articles also observed same effect, showing that pathologic differentiation was associated with OS^13^ and DFS^41^, ^43^ in oral cancer. Kim et al.^25^ have reported pathologic differentiation as an independent factor for OS in patients of oral tongue cancer. Thus, histological grade may be a potential factor indicating the need for postoperative adjuvant therapy, and the patients with a lower degree of differentiation may need more stringent follow‐up after treatment.

The findings of this study should be considered in light of its limitations. Firstly, the retrospective study design leads to inherent biases. Secondly, the TNM staging adopted in this study was based on the 7th but not the currently applied 8th AJCC classification system. Considering that a study involving more than 2000 patients found the upgraded classification was more likely to change stage at T3‐4 and N2,^44^ our adoption of the 7th AJCC edition classification for TSCC patients of T1‐2N0‐1 stage, particularly when 77.5% (341 out of 440) of the patients were in early stage (pT1‐2N0M0), may bear less divergence owing to the choice of the classification edition. Furthermore, the low proportion of cases who received postoperative adjuvant therapy limits the strength of conclusions in this context. In addition, in cases of treatment interruption or loss to follow‐up, which may be caused by patient non‐cooperation, economic limitations, physical tolerance, or other reasons, the outcomes cannot be assessed accurately, and censoring can lead to overestimation of survival rates.^45^ However, this relatively large sampled study analyzing clinical data collected in the short term (2014–2016) with a long follow‐up presents valuable information for translating to clinical practice and research. Future prospective studies with large samples, deeper phenotyping and biomarker data are essential to design improved prognostic indices and precision medicine approaches.

## 5 CONCLUSIONS

This was a single‐center retrospective clinical study with a large sample size and long follow‐up time. The results revealed that the clinical stage of cancer was a crucial factor for outcome after primary resection of TSCC; earlier stages were associated with better prognosis. In addition, poor pathological differentiation emerged as a significant predictor affecting postoperative survival while existence of LVI/PNI was also a prognostic factor. High‐quality LND (≥30) was beneficial for loco‐regional control after surgery for patients with pT1cN0M0 or stage I TSCC, and thus may be recommended for these patients with early‐stage disease to prolong recurrence‐free time.

## AUTHOR CONTRIBUTIONS

**Wenxi Wang:** Data curation (equal); writing – original draft (equal). **Yuxiang Wang:** Formal analysis (equal). **Wenhui Zeng:** Data curation (equal). **Xubin Xie:** Data curation (equal). **Chen Li:** Data curation (equal). **Qin Zhou:** Funding acquisition (equal); project administration (equal). **Liangfang Shen:** Funding acquisition (equal); project administration (equal).

## FUNDING INFORMATION

This work was supported by the National Natural Science Foundation of China (No. 81974466).

## CONFLICT OF INTEREST STATEMENT

The authors declare that they have no competing interests.

## ETHICS STATEMENT

The study protocol was reviewed and approved by the Ethical Review Committee of the Xiangya Hospital Central South University, Hunan, China (202212296).

## CONSENT

This study is a retrospective study, and the data obtained are owned by the medical record itself, so no informed consent was signed.

## Supporting information


Figure S1.

Figure S2.

Table S1.
Table S2.
Table S3.
Table S4.
Table S5.

## Data Availability

The datasets used and/or analyzed during the current study are available from the corresponding author on reasonable request.
