# Supplementary figures and images for: Prognostic factors in surgically treated tongue squamous cell carcinoma in stage T1‐2N0‐1M0: A retrospective analysis
Source: Cancer Med. 2024 Feb 24;13(3):e7016. doi: 10.1002/cam4.7016 (PMC10891452; doi:10.1002/cam4.7016)

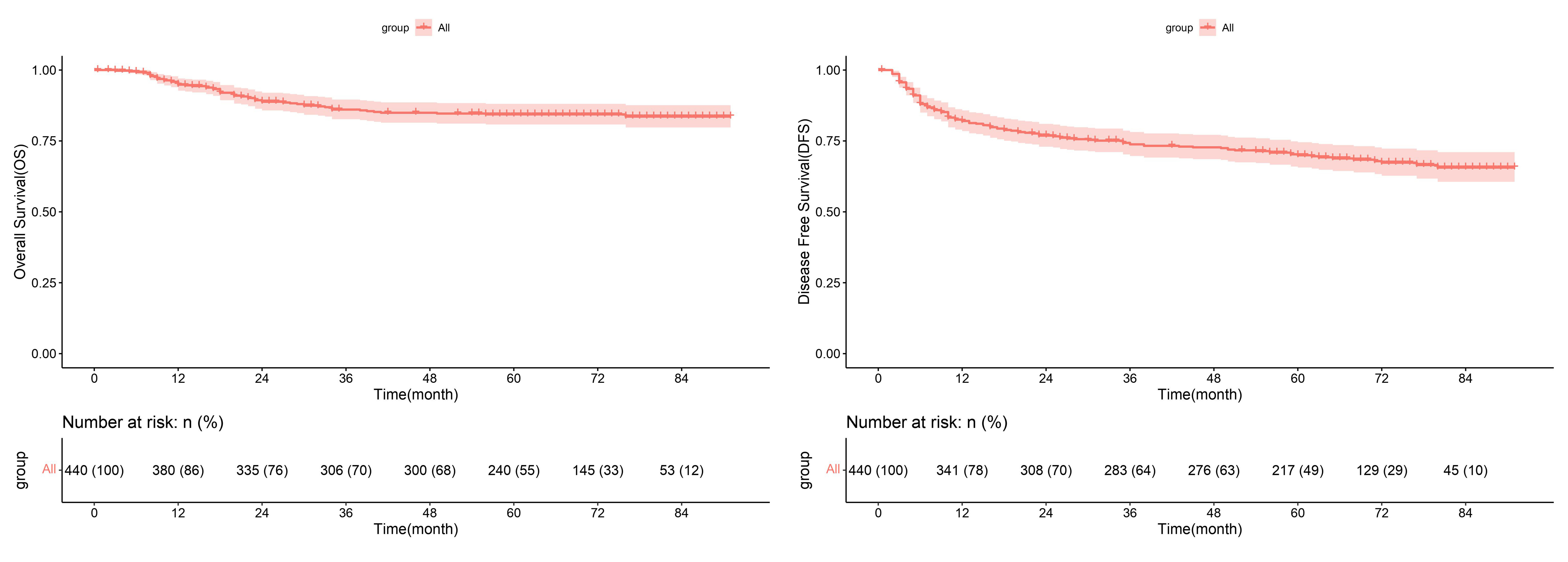

Supplement: Supplementary file 1 — Figure S1. [file CAM4-13-e7016-s001.tif]

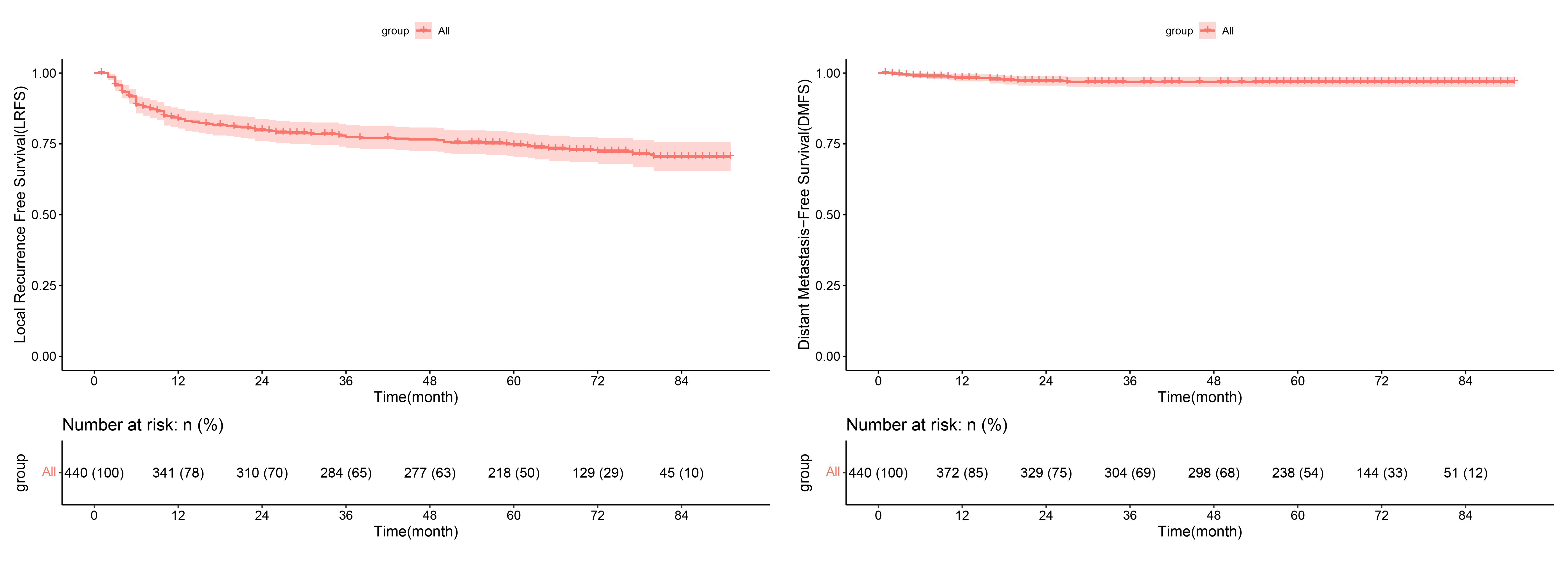

Supplement: Supplementary file 2 — Figure S2. [file CAM4-13-e7016-s002.tif]
