# Supplementary material for: Prognostic factors in surgically treated tongue squamous cell carcinoma in stage T1‐2N0‐1M0: A retrospective analysis
Source: Cancer Med. 2024 Feb 24;13(3):e7016. doi: 10.1002/cam4.7016 (PMC10891452; doi:10.1002/cam4.7016)
Supplement: Supplementary file 3 — Table S1. Table S2. Table S3. Table S4. Table S5. [file CAM4-13-e7016-s003.docx]

**Supplementary table 1.** Univariate COX analysis of factors on OS and DFS in stage pT1-2N0-1M0 patients after surgery

| Items | Adjuvant  therapy | Cases | OS | |  | DFS | |
| --- | --- | --- | --- | --- | --- | --- | --- |
|  |  |  | HR(95%CI) | *P* |  | HR(95%CI) | *P* |
| Gender | Male | 393 | 1 |  |  | 1 |  |
|  | Female | 47 | 1.392(0.686-2.825) | 0.359 |  | 1.087(0.635-1.861) | 0.761 |
| Ages | <50 | 200 | 1 |  |  | 1 |  |
|  | ≥50 | 240 | 1.641(0.961-2.801) | 0.069 |  | 1.041(0.738-1.468) | 0.820 |
| Differ-entiation | Well | 295 | 1 |  |  | 1 | <0.001 |
|  | middle | 129 | 1.512(0.872-2.619) | 0.141 |  | 1.907(1.330-2.733) | <0.001 |
|  | poor | 16 | 4.374(1.836-10.419) | 0.001 |  | 3.352(1.674-6.712) | 0.001 |
| LVI/PNI | No | 391 | 1 |  |  | 1 |  |
|  | Yes | 49 | 2.386(1.268-4.491) | 0.007 |  | 2.101(1.337-3.301) | 0.001 |
| LND | ≥30 | 85 | 1 | 0.11 |  | 1 | 0.087 |
|  | No | 47 | 0.380(0.082-1.760) | 0.216 |  | 2.241(1.152-4.359) | 0.017 |
|  | <15 | 172 | 1.767(0.839-3.722) | 0.134 |  | 1.762(1.007-3.083) | 0.047 |
|  | 15-29 | 136 | 1.534(0.698-3.369) | 0.287 |  | 1.925(1.086-3.413) | 0.025 |
| pT | pT1 | 267 | 1 |  |  | 1 |  |
|  | pT2 | 173 | 2.186(1.321-3.619) | 0.002 |  | 1.507(1.070-2.122) | 0.019 |
| pN | N0 | 391 | 1 |  |  | 1 |  |
|  | N1 | 49 | 2.717(1.631-4.527) | <0.001 |  | 1.613(1.110-2.346) | 0.012 |
| TNM | I | 227 | 1 | <0.001 |  | 1 | 0.008 |
|  | II | 114 | 2.971(1.539-5.735) | 0.001 |  | 1.510(0.997-2.288) | 0.052 |
|  | III | 99 | 4.305(2.298-8.066) | <0.001 |  | 1.864(1.238-2.807) | 0.003 |
| Adjuvant therapy | No | 398 | 1 | 1 |  |  |  |
|  | Yes | 42 | 1.761(0.894-3.469) | 0.102 |  | 1.157(0.676-1.980) | 0.595 |

**Supplementary table 2**. Univariate Cox analysis of factors on LRFS in stage pT1-2N0-1M0 patients after surgery

| Items | Group | HR(95%CI) | P |
| --- | --- | --- | --- |
| Differentiation | Groups |  | <0.001 |
|  | middle | 1.642(1.100-2.451) | 0.015 |
|  | poor | 3.734(1.854-7.520) | <0.001 |
| LVI/PNI | No | 1 |  |
|  | Yes | 1.897(1.143-3.151) | 0.013 |
| LND | ≥30 | 1 | 0.077 |
|  | No | 2.572(1.260-5.250) | 0.009 |
|  | <15 | 1.830(0.987-3.393) | 0.055 |
|  | 15-29 | 1.870(0.989-3.536) | 0.054 |
| pT | pT1 | 1 |  |
|  | pT2 | 1.421(0.978-2.066) | 0.066 |
| pN | N0 | 1 |  |
|  | N1 | 1.350(0.884-2.063) | 0.165 |
| TNM | I | 1 | 0.156 |
|  | II | 1.373(0.879-2.146) | 0.164 |
|  | III | 1.505(0.954-2.375) | 0.079 |
| Adjuvant therapy | No | 1 |  |
|  | Yes | 0.704(0.343-1.445) | 0.339 |

**Supplementary table 3.** Sub-group analysis of LRFS, OS and DFS in postoperative TSCC patients with or without adjuvant therapy

| Stage | Adju-vant  Ther-apy |  | LRFS | | | *P* | OS | | | *P* | DFS | | | *P* |
| --- | --- | --- | --- | --- | --- | --- | --- | --- | --- | --- | --- | --- | --- | --- |
|  |  |  |  |  |  |  |  | | |  |  |  |  |  |
|  |  | Cases | 1-y | 3-y | 5-y |  | 1-y | 3-y | 5-y |  | 1-y | 3-y | 5-y |  |
| pT2 |  |  |  |  |  | 0.150 |  |  |  | 0.934 |  |  |  | 0.599 |
|  | No | 150 | 74.2 | 69.5 | 67.8 |  | 90.8 | 76.8 | 76.8 |  | 72.4 | 65.5 | 63.0 |  |
|  | Yes | 23 | 91.1 | 81.7 | 81.7 |  | 91.3 | 81.9 | 81.9 |  | 87.0 | 73.9 | 69.6 |  |
| N0 |  |  |  |  |  | 0.446 |  |  |  | 0.271 |  |  |  | 0.806 |
|  | No | 333 | 84.8 | 79.4 | 76.0 |  | 95.5 | 89.5 | 88.4 |  | 83.4 | 76.7 | 73.1 |  |
|  | Yes | 8 | 87.5 | 87.5 | 87.5 |  | 100 | 87.5 | 87.5 |  | 87.5 | 87.5 | 87.5 |  |
| N1 |  |  |  |  |  | 0.113 |  |  |  | 0.467 |  |  |  | 0.471 |
|  | No | 65 | 76.0 | 62.6 | 62.6 |  | 93.0 | 71.0 | 66.7 |  | 72.8 | 56.5 | 56.5 |  |
|  | Yes | 34 | 88.0 | 81.1 | 78.1 |  | 90.9 | 78.1 | 74.7 |  | 85.2 | 72.9 | 59.3 |  |

**Supplementary table 4. The OS in pT1 or stage I postoperative TSCC patients with or without LND**

| Items | LND | Cases | OS | | | *P* |
| --- | --- | --- | --- | --- | --- | --- |
|  |  |  | 1-y | 3-y | 5-y |  |
| pT1cN0 |  |  |  |  |  | 0.352 |
|  | No | 41 | 100 | 97.1 | 94.1 |  |
|  | <15 | 99 | 95.6 | 87.1 | 86.0 |  |
|  | 15-29 | 67 | 100 | 93.1 | 91.1 |  |
|  | ≥30 | 36 | 97.0 | 93.8 | 93.8 |  |
| Stage I |  |  |  |  |  | 0.843 |
|  | No | 41 | 100 | 97.1 | 94.1 |  |
|  | <15 | 89 | 97.7 | 90.3 | 90.3 |  |
|  | 15-29 | 70 | 98.4 | 93.5 | 93.5 |  |
|  | ≥30 | 27 | 100 | 96.0 | 96.0 |  |
|  |  |  |  |  |  |  |

**Supplementary table 5.** Pattern of recurrence in pT1cN0 and stage I postoperative TSCC patients with or without LND

|  | LND | Cases | Local | | *P* | Regional | | *P* |
| --- | --- | --- | --- | --- | --- | --- | --- | --- |
|  |  |  | No | Yes |  | No | Yes |  |
| pT1cN0 | No | 41 | 31(75.6%) | 10(24.4%) | 0.250 | 34(82.9%) | 7(17.1%) | 0.009 |
|  | <15 | 99 | 83(83.8%) | 16(16.2%) |  | 91(91.9%) | 8(8.1%) |  |
|  | 15-29 | 67 | 58(86.6%) | 9(13.4%) |  | 57(85.1%) | 10(14.9%) |  |
|  | ≥30 | 36 | 33(91.7%) | 3(8.3%) |  | 36(100%) | 0 |  |
| Stage I | No | 41 | 31(75.6%) | 10(24.4%) | 0.158 | 34(82.9%) | 7(17.1%) | 0.042 |
|  | <15 | 89 | 79(88.8%) | 10(11.2%) |  | 79(88.8%) | 10(11.2%) |  |
|  | 15-29 | 70 | 60(85.7%) | 10(14.3%) |  | 60(85.7%) | 10(14.3%) |  |
|  | ≥30 | 27 | 25(92.6%) | 2(7.4%) |  | 27(100%) | 0 |  |

**Supplementary fig. 1. OS and DFS in patients with stage T1-2N0-1M0 TSCC after surgery**

**Supplementary fig. 2. LRFS and DMFS in patients with stage T1-2N0-1M0 TSCC after surgery**
